# The effect of oxalate-based desensitizers on the microleakage and shear bond strength of resin modified glass ionomer 

**DOI:** 10.4317/jced.52946

**Published:** 2017-10-01

**Authors:** Maryam Firouzmandi, Fereshteh Valipour, Maryam Roshanzamir, Tayebeh Mobaleghi

**Affiliations:** 1 Assistant Professor, Department of Operative Dentistry, School of Dentistry, Shiraz University of Medical Sciences, Shiraz, Iran; 2 Ph.D Student of Pharmaceutical Biomaterial, Faculty of Pharmacy, Tabriz University of Medical Sciences, Tabriz, Iran; 3 Undergraduate Dental Student, Student Research Committee, School of Dentistry, Shiraz University of Medical Sciences,Shiraz, Iran; 4 Postgraduate Student, Department of Periodontics, School of Dentistry, Shahed University of Medical Sciences, Tehran, Iran

## Abstract

**Background:**

The first line of intervention to alleviate tooth sensitivity is to use dentin desensitizers such as oxalate-based desensitizers. When the dentin sensitivity continues even after application of desensitizers the next intervention would be restoration of the lesion. The aim of this *in vitro* study was to investigate the effect of prior application of oxalate-based desensitizer on the marginal microleakage and shear bond strength of resin modified glass ionomer (RMGI) restorations.

**Material and Methods:**

In order to prepare the specimens for microleakage test standard class v cavities were prepared on buccal surfaces of 45 teeth. The specimens were randomly divided into three equal groups. In control group, the cavities were restored with RMGI. In group 2, oxalate-based desensitizer was applied and the specimens were kept in distilled water for 14 days before restoration. In group 3 the specimens were prepared similar to group 2 and the surface of the cavities were slightly cut with bur before restoration. Methyleneblue penetration was evaluated using stereomicroscope. The data were analyzed using non-parametric tests. For shear bond strength test cervical dentin specimens were prepared and were divided into 3 groups. Surface treatments were similar to microleakage test. RMGI was packed into cylindrical plastic molds which were placed on the cut surface of the tooth and light cured. The data were analyzed by one-way ANOVA.

**Results:**

There was not any significant difference in dye penetration in dentin margins among the groups, but microleakage in enamel margins of group 2 and 3 was higher than group 1. There was no significant difference in shear bond strength among the groups (*p* =0.285).

**Conclusions:**

Non carious cervical lesions which were treated for hypersensitivity with oxalate-based desensitizers could be restored with resin modified glass ionomer if the hypersensitivity persists.

** Key words:**Dentin hypersensitivity, oxalate-based desensitizers, microleakage, shear bond strength.

## Introduction

Dentin hypersensitivity is a short, sharp pain which irritates the patients during eating, drinking, brushing, and breathing. The incidence of this condition is ranged between 10-30% (1). Dentin exposure resulting from continuous loss of tooth structure, especially in cervical area, causes dentin hypersensitivity (2). Abrasion, erosion, and abfraction, have all been reported to have crucial effect in the formation of cervical lesions (3). Many theories propounded to explain the mechanism of dentin hypersensitivity, but the hydrodynamic theory is the most broadly accepted. The hydrodynamic theory depicts when thermal, tactile, osmotic, chemical, or evaporative stimuli is applied to dentin, fluid flow is induced in the tubules,therefore, triggering baroreceptors near the pulp and ultimately causing pain for the patient (4).

There are several options for management of the patients who are experiencing dentin hypersensitivity. The underlying mechanism of most of these treatments is to obliterate the dentin tubules. Tubular occlusion can be achieved by desensitizing agents, adhesive systems and restorations (5). The first line of intervention to alleviate tooth sensitivity is to use dentin desensitizers (2). Oxalate-based products showed promising results in reducing dentin sensitivity and permeability (6,7). Potassium oxalate which is the soluble salt of the oxalate reacts with hydroxyapatite in tooth substrate to form insoluble calcium oxalate crystals. These crystals precipitate in the tubules orifices (8). However, there are evidences indicating that mechanical and chemical challenges in the clinical situations may wash out the oxalate precipitates (9). Likewise, potassium oxalate cannot prevent progressive dentin wear. A systematic review on the effect of oxalate in treating dentin hypersensitivity proposed no advantage of oxalate except a placebo effect (10). When the dentin sensitivity continues even after application of desensitizers, and the dentin wear cannot be stopped by elimination of possible etiologic factors; the next intervention would be restoration of the lesion.

Resin modified glass ionomer (RMGI) is one of the restorative materials of choice for restoration of cervical lesions. RMGI has an acceptable bond to enamel and dentin (11), offers high levels of fluoride release, and recharging capacity (12). Because of favorable coefficient of thermal expansion, RMGI results in less microleakage at cervical margins (13). Retention rates of glass ionomer materials are higher than self-etching adhesives in cervical lesions (14,15).

Several studies investigated the effect of oxalate-based desensitizers on adhesion of dental adhesives to dentin (16,17). These studies concluded that oxalate may or may not affect the adhesion to dentin depending on the adhesive used.

The present study aimed to investigate the effect of prior application of an oxalate-based desensitizer onmicroleakage and shear bond strength of RMGI. The null hypotheses to be tested are as follows:

1. Bond strength of RMGI to dentin is not affected by the application of oxalate-based desensitizers and subsequent cutting with bur.

2. Microleakage in cervical cavities restored with RMGI is not affected by the application of oxalate-based desensitizers and subsequent cutting with bur.

## Material and Methods

Following approval of the research protocol by the University Ethics Committee, ninetysix extracted human premolars were collected. The teeth were extracted for orthodontic reasons and were free of any caries, cracks and previous restorations. They were cleaned of any debris, attached soft tissues and calculus, and stored in 4% chloramine-T until use. The teeth were used within one month of extraction. 45 teeth were used for microleakage test, 45 teeth were used for shear bond strength measurement, and six teeth were used for scanning electron microscope (SEM) observation.

-Microleakage test

Standard class V cavities with mesiodistal width of 3 mm, occlusogingival height of 3 mm, and axial depth of 1.5 mm with gingival margin located 1mm below the cementoenamel junction (CEJ) were prepared on buccal surface of teeth using straight diamond burs (# 835/010, teezkavan, Iran) mounted on a high speed hand-piece with air and water cooling. Each bur was discarded after preparation of five cavities.

The specimens were randomly divided into three equal groups (n=15). In group 1(control), polyacrylic acid conditioner (GC Corporation, Tokyo, Japan) was applied to all cavity walls for 20 seconds, rinsed and dried to maintain a moist surface. Then RMGI (Fuji II LC, GC Corporation, Tokyo, Japan) was mixed according to manufacturer’s instructions and packed into the cavity in the increments of 2 mm. Each increment was light cured (Coltolux 75, Whaledent Inc, Coltene, USA)at intensity of 600 mW/cm2 for 30 seconds.

In group 2, the cavities were etched with 35% phosphoric acid (3M ESPE, St Paul, MN, USA) for 15 seconds, rinsed for 10 seconds and gently air dried for 1-2 seconds in a way that the moist condition of the dentin was preserved. Subsequently, oxalate desensitizer (BisBlock, BISCO, Inc, Schaumburg, IL, USA) was applied for 30 seconds and rinsed (16). Then the samples transferred to a container filled with distilled water and kept in an incubator at 37°C for two weeks before restoration. This was done to simulate the time gap between the first line of intervention (desensitizing treatment) and restoration of the lesion. Then the samples were restored using RMGI with the same protocol described for the control group.

In group 3, the specimens were prepared in the same way described for oxalate group but before placing the restoration the cavities were slightly cut for 10 seconds with a cylindrical diamond bur (# 835/010, teezkavan, Iran) to remove oxalate contaminated surface (18), rinsed with water and dried.

All of the specimens were thermocycled (1000 cycles at 5±2ºC/55±2ºC, a 30-second well time, and a 5-second transfer time).

The root apices were sealed with utility wax and all the surfaces, except for the restorations and 1mm from the margins, were coated with two layers of nail varnish. The teeth were immersed in a 0.5% methyleneblue dye solution for 24 hours. They were then rinsed in running water, blot-dried and sectioned longitudinally through the center of the restorations from the facial to lingual surface with a water-cooled diamond wheel saw (Leitz 1600, Wetzlar, Germany). The sections were blindly assessed for dye penetration by two independent evaluators using a stereomicroscope (Carl ZiessInc, Oberkochen, Germany) at 20x magnification. Dye penetration at the composite/tooth interface was scored for both the occlusal and gingival margins on a non-parametric scale from 0 to 3: 0 = no dye penetration; 1 = dye penetration of less than half of the cavity depth; 2 = dye penetration more than half of the cavity depth; 3 = dye penetration spreading along the axial wall. The details of the materials used in this study are provided in  Table 1.

**Table 1:**
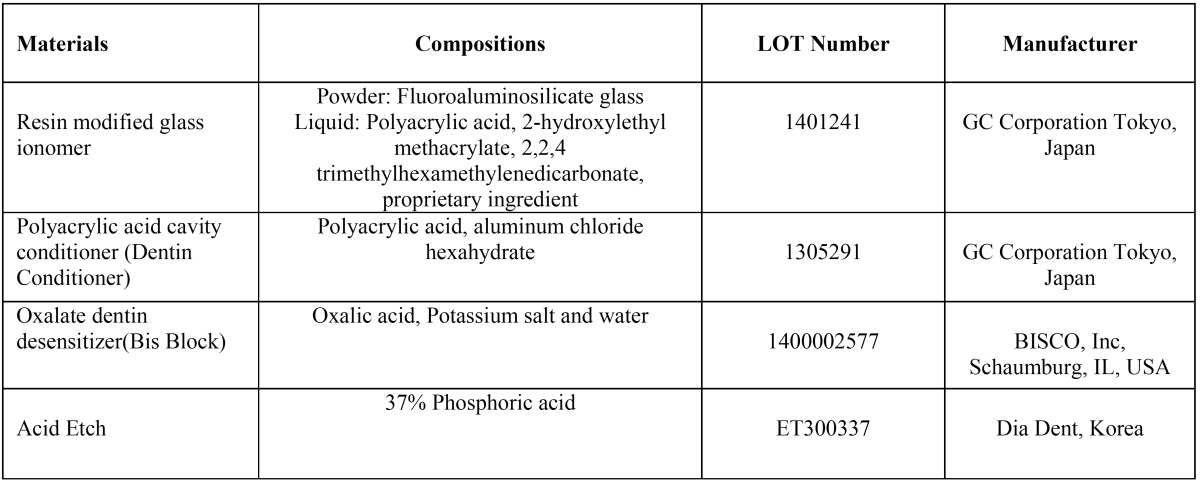
Materials used in this study.

A statistical analysis was performed using the Kruskal-Wallis and Mann-Whitney U-tests (α=0.05).

-Shear bond strength test

Cervical enamel of buccal surfaces of the teeth was grounded with silicon carbide disks(Buehler, USA)under water coolant until the cervical dentin was exposed. The exposed dentin surface was polished with 600 grit sand paper (Carborundum Abrasivos, Recife, PE, Brazil). The remaining coronal and radicular portions were cut and the prepared dentin specimens were mounted in cold cured acrylic resin molds. The specimens were divided into three groups and surface treatments were done following the method described for microleakage test. Subsequently the prepared mixture of Fuji II LC was packed into the cylindrical plastic molds (diameter = 2.38mm, height = 2mm), which were placed on the cut suface of the tooth and light cured. Then the specimens were subjected to thermal cycling (1000 cycles at 5±2ºC/55±2ºC, a 30-second well time, and a 5-second transfer time).

The specimens were tested in shear with a universal testing machine(Zwick-Roell, Zwick, Ulm, Germany)at a cross head speed of 0.5 mm/min. The shear bond strength were calculated and presented in MPa. Debonded surfaces were accessed using stereomicroscope (Carl ZiessInc, Oberkochen, Germany) at 20x magnification. Fracture modes of the specimens were classified as follows: 1. Adhesive, 2. Mixed, 3. Cohesive in RMGI.

The shear bond strength data were analyzed by one-way ANOVA (*p*<0.05). Failure mode data were descriptively reported.

-Scanning electron microscope (SEM) observation

Six dentin specimens (two specimens for each group) were prepared using the method described in shear bond strength test. Subsequent to surface treatments the specimens were dried in a series of ethanol solutions (50%-100%), sputter coated with gold, and observed in high-vacuum condition under SEM (Tescan Vega II, England).

## Results

Dye penetration scores in occlusal and gingival margins are summarized in  Table 2. There was not any significant difference in dye penetration in dentin margins among the groups (*p*=0.347), but microleakage in enamel margins of group 2 and 3 was higher than group 1. *P*-value was 0.006 and 0.018 respectively. There was not any significant difference between group 2 and 3 (*p*=0.177).

**Table 2:**
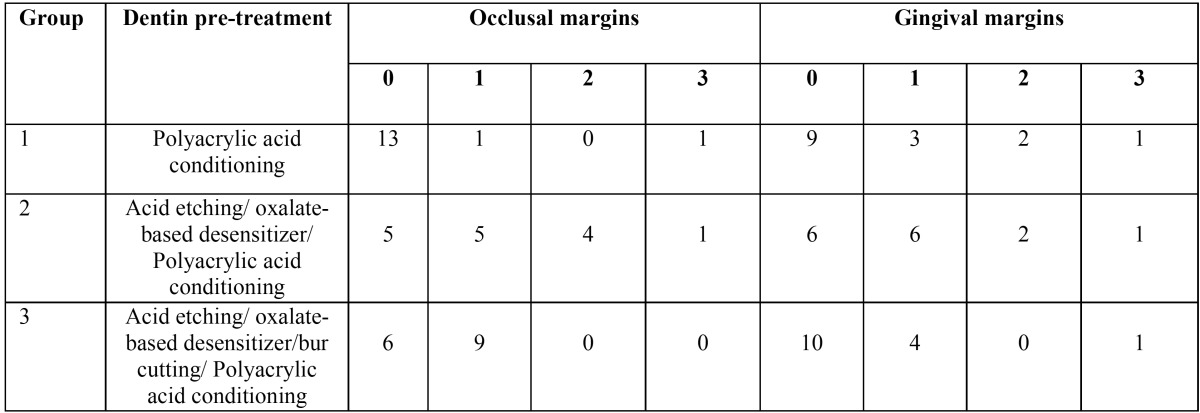
Microleakage scores in occlusal and gingival margins.

The results of the shear bond strength test and failure mode analysis are presented in  Table 3. One-way ANOVA revealed no statistically significant difference between the groups (*p*-Value = 0.285). Predominant failure mode in all three groups was mixed failure.

**Table 3:**
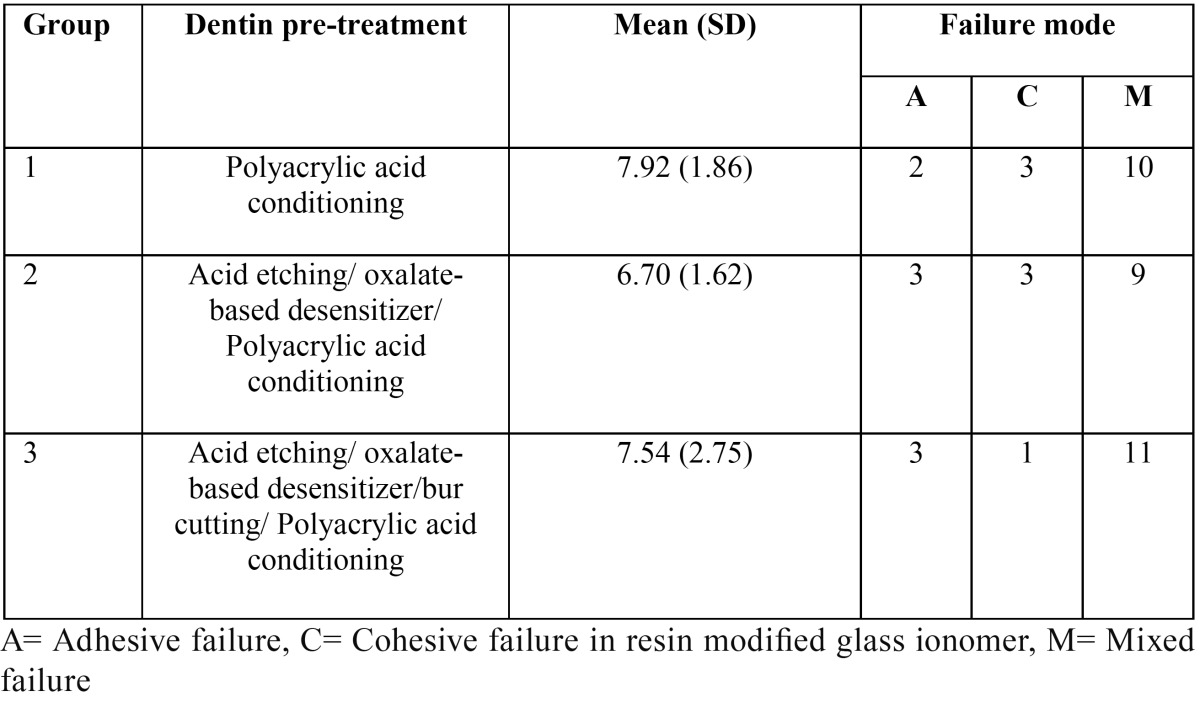
Mean (MPa) and standard deviation (SD) of shear bond strength and failure modes in the study groups.

**Figure 1:**
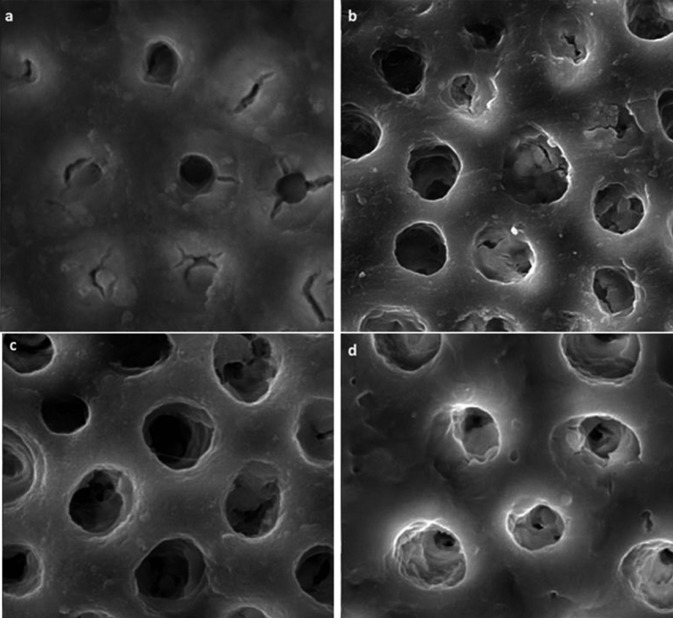
Representative scanning electron microscopy (SEM) images of dentin surface following different treatment methods: (a) conditioned with polyacrylic acid, (b) etched with 37% phosphoric acid and treated with oxalate-based desensitizer, (c) etched with 37% phosphoric acid, treated with oxalate-based desensitizer, and conditioned with polyacrylic acid, (d) etched with 37% phosphoric acid, treated with oxalate-based desensitizer, slightly cut with bur, and conditioned with polyacrylic acid .

SEM analysis demonstrated that polyacrylic acid conditioned dentin surface was covered with smear layer, and the tubules were occluded with smear plug (Fig. 1a). The tubules in acid-etched oxalate-treated dentin were funnel shape and obstructed with precipitates. As it is appeared in figure 1b precipitates formed deep inside the tubules. Subsequent polyacrylic acid conditioning dissolved some of the precipitates and results in more patent tubules (Fig. 1c). Although slight cutting with bur removed the superfacial layer the tubules remained obstructed (Fig. 1d).

## Discussion

According to the results of the shear bond strength test in the present study, bond strength of RMGI to dentin in the control group was not significantly different from the study groups. Microleakage study also revealed that there wasn’t any significant difference in dye penetration in dentin margins among the groups. So the null hypothesis was accepted inpart. However, microleakage in enamel margins of the control group was lower than the study groups.

The effect of different conditioning methods on adhesion of RMGI to dentin has been investigated (19,20). Among these conditioners, polyacrylicacid let out the most favorable results(19,21). That is why it is recommended by the manufacturer of Fuji II LC. Since RMGI can bond to unground dentin according to the manufacturer’s instructions and mechanical retention form is not requisite for them, there is no need to prepare the tooth before restoration in many non-carious cervical lesion (NCCL) cases (15). In group 2 RMGI was bonded without any preparation, and in group 3 a diamond bur was employed for a brief cutting to remove oxalate contamination, similar to the method used by a previous study to remove surface contaminations (18).

The data obtained from bond strength tests are notconclusive enough to predict the efficacy of restorative materials in clinical situations; compared to resin composite, better performance of RMGI in NCCL would possibly demonstrates this disparity (14,15,22). Microleakage tests can be used as a complementary tool for bond strength tests.

The results of the current study showed that application ofoxalate-based BisBlock (BiscoInc;Schaumburg, IL, USA) desensitizer on acid etched dentin did affectneither the adhesion of RMGI to dentin nor the microleakage of dentin margins. The adhesion of RMGI to dentin includes chemical bonds and micromechanical interlockings. Ionic interaction between the carboxyl groups of the polyalkenoic acid and calcium of hydroxyapatite results in true primary chemical bonding (23). Micromechanical entanglement as the second part of the dual adhesive mechanism occurs through the formation of a hybrid-like layer. Resin monomers included in the formulation of Fuji II LC infiltrates into the porous collagen network of conditioned dentin creating an interdiffusion zone (11,24).

The etching step before oxalate application dissolves calcium ions of smear layer and underlying dentin. Therefore, calcium oxalate crystals tend to form in the tubules leaving the dentin surface ready for resin infiltration (Fig. 1b) (25,26). Acid phosphoric etching compromises the potential of ionic bonding to the mineral component of the tooth (27). However, subsequent oxalate treatment creates a layer of crystalline precipitates rich in both calcium and carboxylate groups which might promote chemical bonding (28).

The stability of calcium oxalate crystals is related to the pH of the environment (16,29). In the present study cavity conditioner used before RMGI bonding has an acidic pH of 0.97. This could be neutralized by calcium oxalate crystals. As it is appeared in figure 1c cavity conditioner might dissolve calcium oxalate precipitates to some extent. Consequently free calcium ions become available to interact with carboxyl groups and contribute to the chemical bonding between RMGI and tooth structure. Dissolution of calcium oxalate would also be enhanced by the fluoride available in composition of RMGI (8,29).

Oxalate crystals forms 10-15 µm beneath the dentin surface (9,25). Slight cutting of the cavity surface with bur should not com-pletely remove this interaction zone (Fig. 1d) (18).

Therefore, the opportunity for chemical interactions is preserved. Besides, polyacrylic acid conditioning might also expose some more collagen for micromechanical entanglement.

Microleakage of oxalate-treated enamel margins increased, and even slight cutting of the surface with bur could not eliminate the effect of oxalate. Calcium oxalate crystals formed on enamel surface can interfere with resin infiltration because the interaction of potassium oxalate with enamelis superficial compared to dentin (25).

Slight cutting of the oxalate-treated enamel surface with bur might result in formation of smear layer rich in calcium oxalate crystals. Fuji II LC conditioner might fail to remove this acid-resistant smear layer. Therefore micromechanical interlocking ofRMGI to enamel is compromised.

Failure mode analysis revealed that mixed failures was predominant in all three groups. This finding is attributed to the weak cohesive strength of RMGI. Bond strength values obtained in the present study might be limited by cohesive strength of RMGI.

In the current study the teeth were kept in distilled water instead of artificial saliva after oxalate treatment and before being bonded with FujiII LC. This might be considered as a limitation of this study. It is proposed that calcium oxalate may slowly be dissolved in artificial saliva (30). Furthermore, chemical and mechanical challenges of the oral environment such as acidic erosion and tooth brushing abrasion are not simulated.

Different chemical composition of other RMGIs might give rise to variations in adhesion mechanism. Future studies are suggested to investigate the effect of oxalate containing desensitizers on bonding performance of different types of RMGIs.

According to the results obtained in the present study it might be concluded that:

1. Oxalate-baseddesensitizers do not adversely affect the microleakage in dentin-RMGI interface.

2. Oxalate-based desensitizers increase the microleakage in enamel-RMGI interface.

3. Bond strength of RMGI to dentin is not influenced by prior application of oxalate-based desensitizers.

4. Slight cutting of the oxalate-treated dentin surface with bur does notinfluence the bond strength of RMGI to dentin.
